# Mutation-agnostic RNA interference with engineered replacement rescues *Tmc1*-related hearing loss

**DOI:** 10.26508/lsa.202201592

**Published:** 2022-12-27

**Authors:** Yoichiro Iwasa, Miles J Klimara, Hidekane Yoshimura, William D Walls, Ryotaro Omichi, Cody A West, Seiji B Shibata, Paul T Ranum, Richard JH Smith

**Affiliations:** 1 Department of Otorhinolaryngology, Shinshu University School of Medicine, Matsumoto, Japan; 2 Molecular Otolaryngology and Renal Research Laboratories, Department of Otolaryngology – Head and Neck Surgery, University of Iowa, Iowa City, IA, USA; 3 Department of Otolaryngology – Head and Neck Surgery, Okayama University Graduate School of Medicine, Dentistry and Pharmaceutical Sciences, Okayama, Japan; 4 Department of Otolaryngology Head and Neck Surgery, Keck School of Medicine of USC, University of Southern California, Los Angeles, CA, USA; 5 Raymond G. Perelman Center for Cellular and Molecular Therapeutics, The Children’s Hospital of Philadelphia Research Institute, Philadelphia, PA, USA

## Abstract

Hearing loss is the most common sensory deficit, of which genetic etiologies are a frequent cause. Dominant and recessive mutations in *TMC1*, a gene encoding the pore-forming subunit of the hair cell mechanotransduction channel, cause DFNA36 and DFNB7/11, respectively, accounting for ∼2% of genetic hearing loss. Previous work has established the efficacy of mutation-targeted RNAi in treatment of murine models of autosomal dominant non-syndromic deafness. However, application of such approaches is limited by the infeasibility of development and validation of novel constructs for each variant. We developed an allele-non-specific approach consisting of mutation-agnostic RNAi suppression of both mutant and WT alleles, co-delivered with a knockdown-resistant engineered WT allele with or without the use of woodchuck hepatitis virus post-transcriptional regulatory element (WPRE) to augment transgene expression. This therapeutic construct was delivered into the mature murine model of DFNA36 with an AAV vector and achieved robust hair cell and auditory brainstem response preservation. However, WPRE-enhanced *Tmc1* expression resulted in inferior outcomes, suggesting a role for gene dosage optimization in future *TMC1* gene therapy development.

## Abstract (precis)

Mutation-agnostic RNA interference with concomitant, knockdown-resistant gene replacement, results in robust auditory brainstem response and cochlear hair cell preservation in the murine model of *TMC1*-related autosomal dominant hearing loss.

## Introduction

Hearing loss is the most common sensory deficit. It impacts one of every 500 newborns and in at least 50% of cases is genetic in etiology (1). Current therapies for genetic hearing loss such as cochlear implants and hearing aids are symptomatic in nature and do not restore normal auditory function (2). The advent of precision medicine, next-generation sequencing technologies, and the widespread adoption of targeted genetic testing panels has resulted in unprecedented access to molecular diagnosis and growth in understanding the diverse genetic etiologies of hearing loss. This knowledge, in turn, has spurred research in potentially curative treatments such as hair cell regeneration and gene-specific therapies.

Autosomal dominant non-syndromic hearing loss arises because of pathogenic variants with dominant-negative, gain-of-function, or haploinsufficiency mechanisms of action, and accounts for ∼15–20% of genetic hearing loss diagnoses (3, 4). Several cochlear gene therapies have leveraged adeno-associated virus (AAV) to deliver RNAi or CRISPR/Cas9 constructs to reduce or rescue hearing impairment in neonatal mouse models of autosomal dominant non-syndromic hearing loss (5, 6, 7). Although these treatments represent substantial advancements toward the clinical application of gene therapy to address hearing loss, delivery before the onset of murine hearing at ∼P12–P15 limits direct applicability to the human cochlea, which is mature at birth (8, 9, 10).

*TMC1* encodes transmembrane channel–like protein 1, the pore-forming subunit of the mechanotransduction complex required for conversion of the intracochlear pressure wave to an electrical signal (11). Dominant-negative and loss-of-function variants in *TMC1* cause DFNA36 and DFNB7/11, respectively, which comprise ∼2% of genetic hearing loss diagnoses (3). Both dominant and recessive mouse models of *TMC1*-related deafness have been successfully treated with gene therapies (9, 10, 12, 13, 14, 15, 16). The *Beethoven* (*Bth*) mouse is a well-described murine model of DFNA36, which carries the semi-dominant c.1235T > A (M412K) mutation in *Tmc1*, resulting in a progressive hearing loss phenotype similar to the post-lingual hearing loss seen in humans segregating the orthologous M418K in *TMC1* (17). We previously reported the efficacy of mutation-specific RNAi-based gene therapy using an AAV2/9 vector in neonatal and mature *Tmc1*^*Bth/+*^ mice (6, 10).

**Figure 1.**
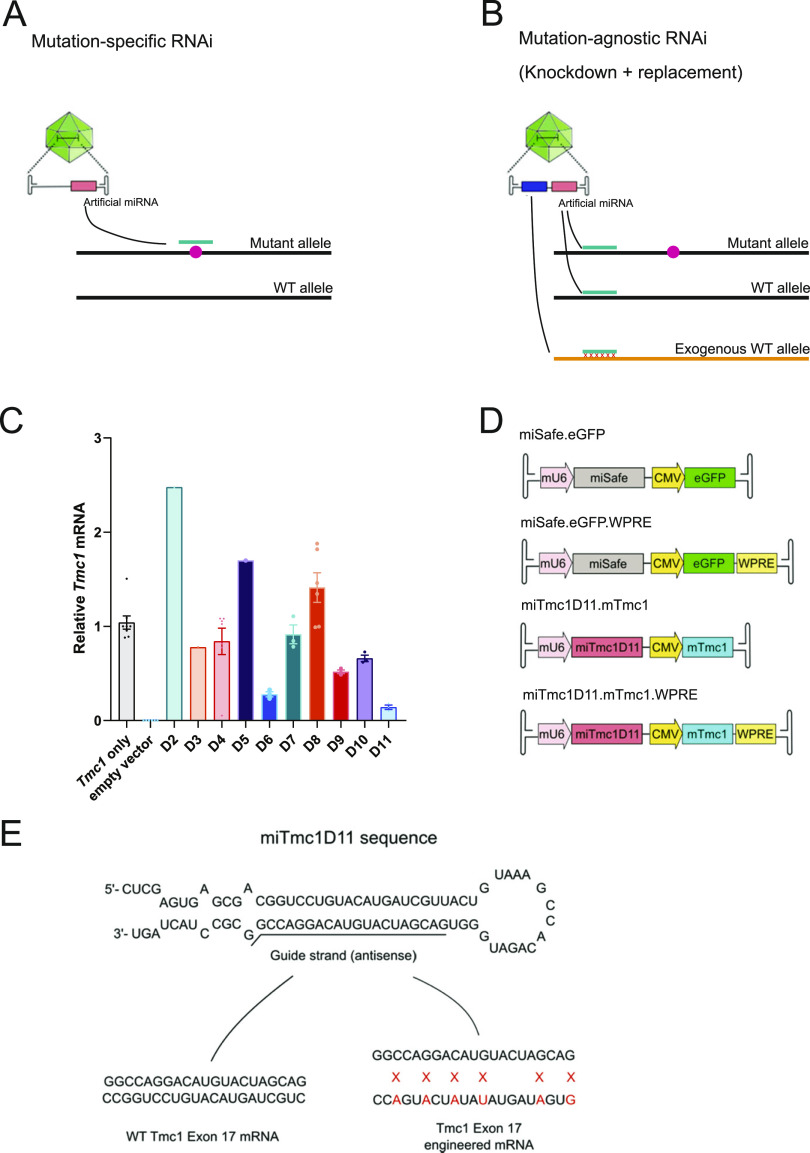
Overview of therapeutic outline, in vitro knockdown efficiency of miTmc1D11, and gene therapy constructs. **(A)** Illustration of mutation-specific RNAi, in which a targeted RNAi construct suppresses a mutant allele. **(B)** Illustration of mutation-agnostic RNAi, wherein the vector delivers an RNAi construct to suppress endogenous (WT and mutant) transcripts of the gene, and a knockdown-resistant exogenous WT allele, which is engineered to have synonymous variants at the miRNA-targeting site. **(C)** qRT-PCR analysis isolated from COS-7 cells co-transfected with WT *Tmc1* and either miTmc1D02-D11 or empty vector. Knockdown efficiency of miTmc1D11 was >80%. Data are the mean ± SEM, and each point represents a single biological replicate, the average of three technical triplicates. **(D)** Outline of transgene control and gene therapy constructs. The mU6 promoter drives miSafe or miTmc1D11 expression, and CMV promoter drives expression of eGFP or engineered *Tmc1* cDNA ± WPRE. **(E)** miTmc1D11 sequence and diagram of knockdown-resistant exogenous *Tmc1*. Exogenous WT mouse *Tmc1* has been engineered to have six synonymous variants in exon 17 to escape RNAi suppression.

Traditional RNAi-based gene therapy, however, has several limitations. First, mutation-specific RNAi therapeutics (Fig 1A) must be independently developed and validated for each causative variant. Allele-non-specific RNAi, in which both WT and mutant RNA transcripts are suppressed, and exon-specific strategies are proposed as potential methods to circumvent this limitation (18). However, this approach alone is only suitable for genes in which knockdown of the WT allele or exon does not result in disease. Second, RNAi-based gene therapy is primarily applicable to dominant-negative forms of hearing loss, whereas genetic hearing loss exhibits multiple modes of inheritance, of which recessive inheritance because of biallelic loss-of-function variants is the most common pattern. To overcome inherent limitations of mutation-targeted RNAi strategies, Kiang et al proposed a mutation-agnostic strategy involving RNAi suppression of both WT and mutant transcripts, with simultaneous delivery of RNAi-resistant engineered gene replacement (Fig 1B) (19). This approach has since been successfully deployed in animal models of systemic and sensorineural diseases including α-1 antitrypsin deficiency and retinitis pigmentosa (20, 21).

We have extended this technique to design and optimize therapeutic AAV vectors, which deliver both an RNAi construct and a custom-engineered, knockdown-resistant WT gene expression construct. The siRNA sequence targets a position present on both WT and mutant alleles of the endogenous mRNA, rather than a specific site carrying the causative mutation. An engineered WT cDNA construct altered by the inclusion of synonymous variants at the siRNA-binding site is included to escape RNAi gene silencing. This therapeutic strategy may be broadly applicable to other forms of hearing loss regardless of the mode of inheritance and mechanism of disease. Herein, we report the first use of this strategy in a mature murine model of *TMC1*-related hearing loss.

## Results

### Design of mutation-agnostic RNAi with gene replacement strategy

#### Tmc1 siRNA and engineered gene replacement design

**Figure S1.**
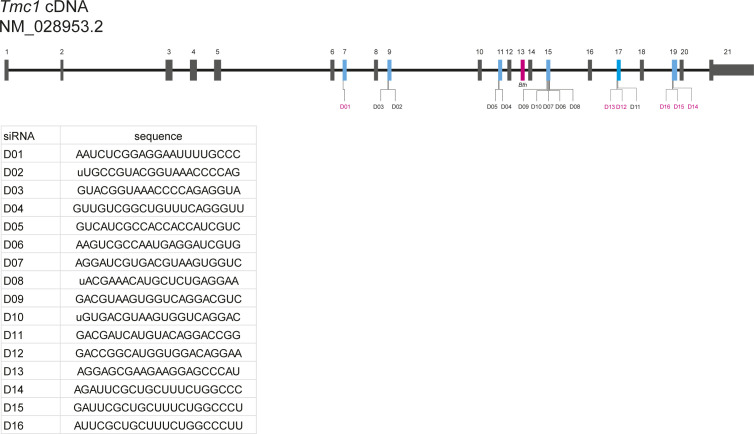
*Tmc1* cDNA structure and siRNAs evaluated in this study. Schematic illustration of *Tmc1* exonic structure, illustrating the position of siRNAs designed in this study. Blue exons were targeted by siRNAs designed by siSPOTR. siRNAs labeled in magenta were not successfully cloned into the pFBAAVmU6mcsCMVeGFP expression plasmid and tested for knockdown efficiency with RT–PCR. Inset: siRNA sequences.

**Figure S2.**
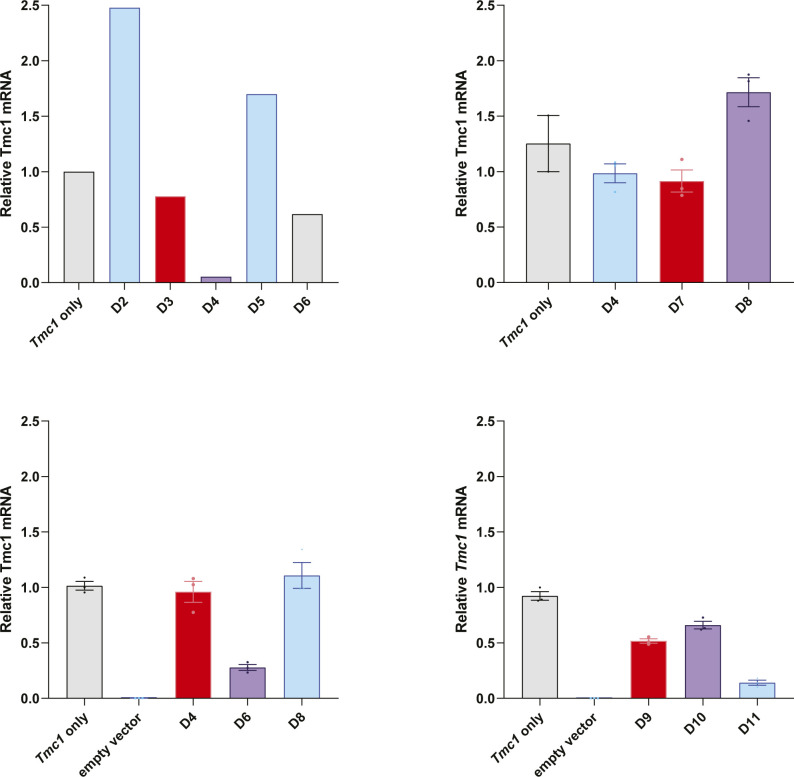
Results of individual RT–PCR experiments. Knockdown efficiency of D02–D11 across four RT–PCR experiments, which are summarized in Fig 1C. Data are means ± SEM with each point representing a single biological replicate, the average of three technical triplicates.

siRNAs that do not target the sequence including the M412K mutation were designed to target exons 7, 9, 11, 15, 17, and 19 of the endogenous mouse *Tmc1* mRNA (NM_028953.2) using siSPOTR (Fig S1) (22). The miRNA hairpins were prioritized based on predicted binding affinity and potential off-target score and cloned into the multiple cloning site of the pFBAAVmU6mcsCMVeGFP expression plasmid obtained from the University of Iowa Viral Vector Core Facility. Knockdown efficiency was tested in vitro in COS-7 cells, a *Tmc1*-deficient cell line, by co-transfection of *Tmc1* and miRNA D02-11 expression plasmids followed by qRT-PCR. miRNA D11 (miTmc1D11) resulted in the highest in vitro knockdown efficiency (>80%) relative to empty vector controls (Fig 1C; knockdown efficiency across contributing individual experiments is summarized in Fig S2). miTmc1D11 was therefore selected for inclusion in the final viral vector design.

miTmc1D11 driven by the mU6 promoter and the *Tmc1* gene replacement expression construct driven by the CMV promoter were combined into a single plasmid vector (Fig 1D). *Tmc1* cDNA (referred to as mTmc1) was engineered to have synonymous variants at degenerative wobble positions within the miTmc1D11-targeting site in exon 17 to escape RNAi suppression (Fig 1E).

#### Transduction efficiency and auditory outcomes with control vectors

**Figure 2.**
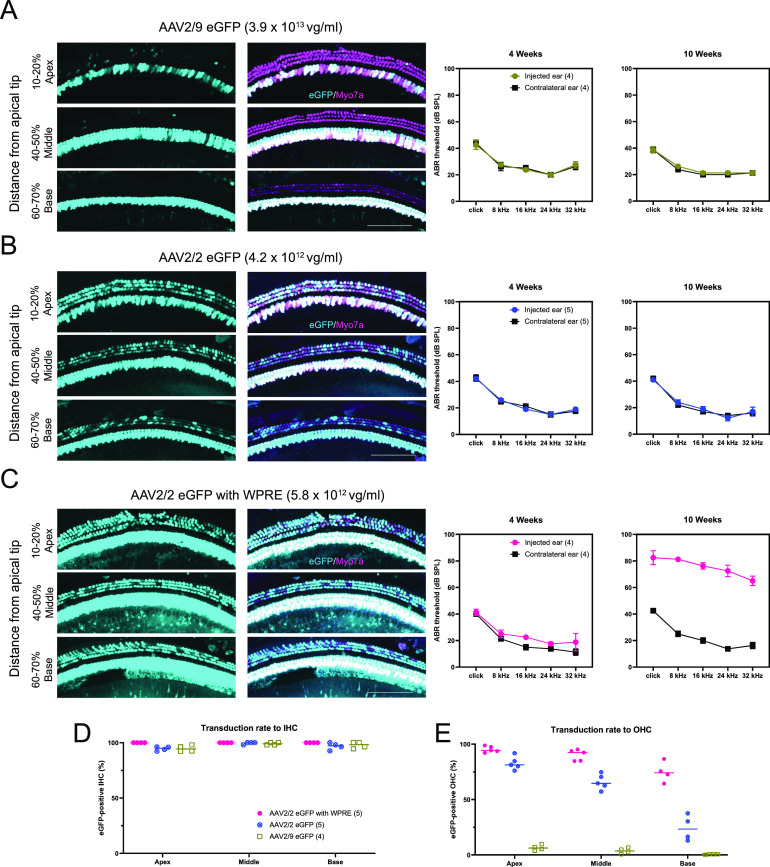
Transduction efficiency and auditory brainstem response after eGFP expression viral vector injection. **(A, B, C)** 2 wk after injection of eGFP expression viral vectors, whole-mount dissection was performed. Cochleae were stained with Myo7a (magenta) for labeling hair cells and imaged for native eGFP (cyan). Auditory brainstem responses were measured in the injected ear and uninjected contralateral ear at 4 and 10 wk of age. Only AAV2/2 eGFP w/WPRE-injected mice showed hearing impairment. Scale bar represents 100 μm. **(D, E)** Transduction efficiency in inner hair cells and outer hair cells. Data are means ± SEM. *N* mice per experimental condition are in parentheses.

Transduction efficiency was evaluated after round window membrane with canal fenestration (RWM + CF) injection at P18 by quantifying eGFP expression in whole-mount preparations of the membranous labyrinth at 4 wk of age, after injection of AAV2/9.miSafe.eGFP (Fig 2A), AAV2/2.miSafe.eGFP (Fig 2B), and AAV2/2.miSafe.eGFP.WPRE (woodchuck hepatitis virus post-transcriptional regulatory element) (Fig 2C) in C3HeB/FeJ (C3H) WT mice. miSafe is an arbitrary sequence with low off-targeting potential, and WPRE is a *cis*-acting post-transcriptional regulator that increases transgene expression up to fivefold in a tissue-specific fashion (23, 24). For readability, these WT control groups will be referred to as AAV2/9 eGFP, AAV2/2 eGFP, and AAV2/2 eGFP w/WPRE, respectively (Table 1).

**Table 1.** Overview of experimental groups described in this study

Group name	*Tmc1* genotype	Viral vector name	Titer (vg/ml)
WT control	*+/+*	None	n/a
AAV2/9 eGFP	*+/+*	AAV2/9.miSafe.eGFP	3.9 × 10^13^
AAV2/2 eGFP	*+/+*	AAV2/2.miSafe.eGFP	4.2 × 10^12^
AAV2/2 eGFP w/WPRE	*+/+*	AAV2/2.miSafe.eGFP.WPRE	5.8 × 10^12^
*Bth/+* control	*Bth/+*	None	n/a
AAV2/9 replacement alone	*Bth/+*	AAV2/9.miSafe.mTmc1	1.8× 10^13^
AAV2/9 RNAi + replacement	*Bth/+*	AAV2/9.miTmc1D11.mTmc1	2.1 × 10^13^
AAV2/2 RNAi + replacement	*Bth/+*	AAV2/2.miTmc1D11.mTmc1	4.0 × 10^12^
AAV2/2 RNAi + replacement w/WPRE	*Bth/+*	AAV2/2.miTmc1D11.mTmc1.WPRE	4.8 × 10^12^
*Bth/+* + AAV2/2 eGFP	*Bth/+*	AAV2/2.miSafe.eGFP	4.2 × 10^12^

For readability, a shorthand description of each experimental group is used throughout this study.

**Figure S3.**
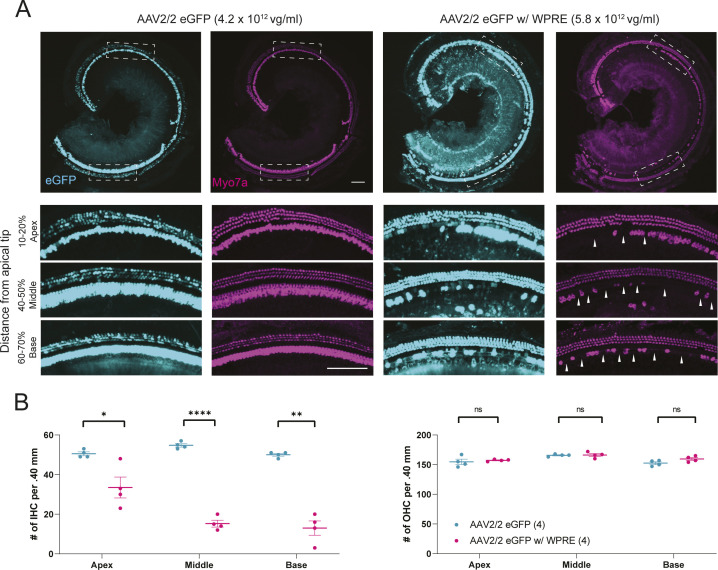
Immunohistochemistry at 10 wk of age after RWM + CF inoculation with eGFP marker vectors. **(A)** Whole-mount immunostaining of WT controls that received AAV2/2 eGFP and AAV2/2 eGFP w/WPRE. Whole-mount dissection and immunostaining for Myo7a (magenta) and imaging for native eGFP (cyan) were performed at 10 wk of age. Scale bars represent 100 μm, and white dashed boxes represent regions of high-magnification image capture for cell counting. Arrowheads: hair cell loss. **(B)** Cell counting of WT controls that received marker vectors. AAV2/2.miSafe.eGFP-injected mice showed no evidence of hair cell loss. AAV2/2.miSafe.eGFP.WPRE-injected mice showed hair cell loss at 10 wk of age, consistent with the observed increase in auditory thresholds (Fig 2C). Hair cell loss was predominantly observed in inner hair cells, whereas outer hair cells were intact. Data are means ± SEM. *N* mice per experimental condition are indicated in parentheses. Statistical analysis was performed by Welch’s *t* test. Inner hair cell comparisons: apex, *P* = 0.046; middle, *P* < 0.0001; and base, *P* = 0.002. ****P < 0.0001; **P < 0.01; and *P < 0.05.

All vectors tested resulted in ≥94.5% transduction of inner hair cells (IHCs) throughout all turns of the cochlea (Fig 2D). However, the transduction of outer hair cells (OHCs) varied by AAV serotype and across the tonotopic axis: AAV2/9 eGFP transduced 6.5% of OHCs in the apical turn and 0.5% of OHCs within the basal turn, whereas AAV2/2 eGFP transduced 82.8% and 24.3% of OHCs within the apical and basal turns, respectively (Fig 2E). AAV2/2 eGFP w/WPRE resulted in increased transduction efficiency, with 74.8% of OHCs in the basal turn expressing eGFP and eGFP fluorescence detectable within the spiral ganglion region (Fig 2C and E). No hair cell loss was seen at 4 wk in any mice regardless of the type of injected vectors, suggesting that none of the vectors trialed was immediately damaging to the cochlea. At 10 wk of age, loss of 34–74% of IHCs was observed only with AAV2/2 eGFP w/WPRE; no OHC loss was observed (Fig S3).

Auditory brainstem response (ABR) recordings were obtained at 4 and 10 wk of age after P18 RWM + CF injection. At 4 wk, AAV2/9 eGFP- and AAV2/2 eGFP-injected control mice showed no evidence of hearing impairment because of viral injection (Fig 2A and B). AAV2/2 eGFP w/ WPRE treatment resulted in a slight, variable threshold elevation of 5–15 dB compared with uninjected contralateral ears (Fig 2C). At 10 wk, AAV2/9 eGFP and AAV2/2 eGFP treatment resulted in no hearing impairment (Fig 2A and B), whereas ears treated with AAV2/2 eGFP w/ WPRE exhibited severe hearing loss (Fig 2C). Based on these results, both AAV2/2 and AAV2/9 serotypes were selected for packaging of the therapeutic constructs.

### Gene therapy prevents progression of hearing loss in *Bth*/+ mice

**Figure 3.**
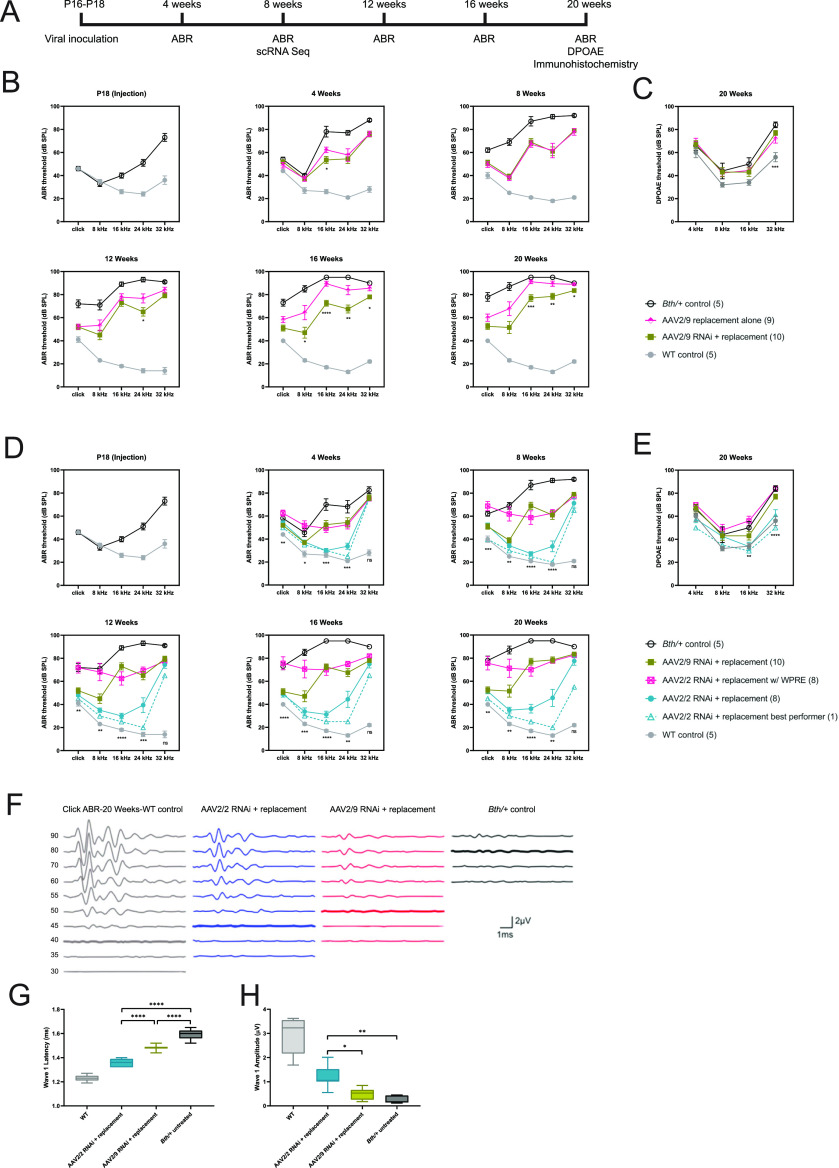
Allele-non-specific RNAi with engineered replacement durably improves auditory performance in *Tmc1*^*Bth/+*^ mice. **(A)** After viral inoculation at P16–18, auditory brainstem responses (ABRs) were measured every 4 wk until 20 wk of age. DPOAE and immunohistochemistry were conducted at 20 wk. Hair cells were extracted for single-cell RNA-Seq at 8 wk. Statistical testing for ABR and DPOAE thresholds is reported in Tables S1–S4. **(B)** ABR thresholds recorded from 4 to 20 wk in *Bth/+* mice treated with AAV2/9 gene replacement alone and AAV2/9 RNAi + gene replacement. **(C)** DPOAE thresholds recorded at 20 wk. **(D)** ABR thresholds recorded from 4 to 20 wk in *Bth/+* mice treated with AAV2/9 RNAi + replacement, replacement, and AAV2/2 RNAi + AAV2/2 RNAi +replacement with WPRE. **(E)** DPOAE thresholds recorded at 20 wk. **(F)** Representative click ABR waveforms at 20 wk of age. **(G, H)** Wave 1 latency (G) and amplitude (H) at 90 dB SPL of WT control, AAV2/2 RNAi + replacement, AAV2/9 RNAi + replacement, and *Bth/+* control were compared. Latency comparison: one-way ANOVA, *P* < 0.0001, and Tukey’s multiple comparisons test, *P* < 0.0001 for all between-group comparisons; amplitude comparison: Welch’s ANOVA, *P* < 0.0001, and Dunnett’s T3 multiple comparisons test: *P* = 0.003 for AAV2/2 RNAi + replacement versus *Bth/+* control, *P* = 0.02 for AAV2/2 RNAi + replacement versus AAV2/9 RNAi + replacement, and *P* = 0.2 for AAV2/9 RNAi + replacement versus *Bth/+* control. ****P < 0.0001; ***P < 0.001; **P < 0.01; and *P < 0.05. Data are means ± SEM. *N* mice per experimental condition are indicated in parentheses.

Viral vector was injected at P16–18, and ABR thresholds were measured in response to click and tone-burst stimuli every 4 wk in the following groups (Fig 3A; summary of the seven experimental groups is shown in Table 1): (1) WT controls, (2) untreated *Tmc1*^*Bth/+*^ controls, and *Tmc1*^*Bth/+*^ mice that received the following treatments: (3) AAV2/9 gene replacement alone, (4) AAV2/9 RNAi + gene replacement, (5) AAV2/2 RNAi + gene replacement, (6) AAV2/2 RNAi + replacement with WPRE, and (7) *Bth/+* + AAV2/2 eGFP.

Table S1 Summary statistical analysis (mean and Welch’s *t* test *P*-value) of mean auditory brainstem response thresholds of animals treated with AAV2/9 replacement alone versus AAV2/9 RNAi + replacement as summarized in Fig 3B.

Table S2 Summary statistical analysis (mean and *P*-values) of DPOAE threshold of animals treated with AAV2/9 replacement alone versus AAV2/9 RNAi + replacement versus WT and *Bth/+* control as summarized in Fig 3C.

Table S3 Summary statistical analysis (mean and *P*-values) of auditory brainstem response thresholds of animals treated with AAV2/9 RNAi + replacement versus AAV2/2 RNAi + replacement versus AAV2/2 RNAi + replacement w/ WPRE as summarized in Fig 3D.

Table S4 Summary statistical analysis (mean and *P*-values) of DPOAE thresholds of animals treated with AAV2/9 RNAi + replacement versus AAV2/2 RNAi + replacement versus AAV2/2 RNAi + replacement w/WPRE versus untreated WT and *Bth/+* controls as summarized in Fig 3E.

*Bth/+* controls exhibited hearing loss that progressed to profound levels by 20 wk of age, consistent with prior reports (5, 8, 15). In light of previous successful rescue with an allele-specific strategy using the AAV2/9 serotype (6), rescue of mouse models of DFNB7/11 using gene replacement, and partial preservation of auditory function in *Tmc1*^*Bth/+*^ mice with *Tmc2* gene replacement alone (12, 14), we compared treatment efficacy of AAV2/9.miSafe.mTmc1 with AAV2/9.miTmc1D11.mTmc1 to test the efficacy of *Tmc1* expression augmentation (gene replacement alone) and with RNAi + gene replacement (Fig 3B). Until 12 wk of age, thresholds were comparable between mice that received injections with AAV2/9 replacement alone and AAV2/9 RNAi + replacement, with both groups exhibiting mean click ABR thresholds 25 dB better than *Bth/+* controls. After 16 wk of age, thresholds of mice that received AAV2/9 gene replacement alone exhibited significantly worse hearing than AAV2/9 RNAi + replacement across mid- and high frequencies. By 20 wk of age, click and 8-kHz tone-pip ABR thresholds were ∼10–20 dB better in AAV2/9 RNAi + replacement relative to AAV2/9 replacement alone, and ∼40 dB better relative to untreated *Bth/+* controls. Treatment efficacy was limited at 16–32 kHz but was significantly better with RNAi + replacement than with replacement alone. Regardless of therapeutics, DPOAE thresholds of all *Bth/+* animals were significantly elevated related to WT controls; DPOAE thresholds did not differ among AAV2/9 replacement alone, AAV2/9 RNAi + replacement, and untreated *Bth/+* mice at 20 wk (Fig 3C).

We hypothesized that improved transduction of OHCs and increased transgene expression might further improve ABR and DPOAE outcomes. We compared ABR thresholds of AAV2/9 RNAi + replacement, AAV2/2 RNAi + replacement, and AAV2/2 RNA + replacement with WPRE at each tested time point to evaluate the difference between AAV2/2 and AAV2/9, and with the use of the WPRE to enhance transgene expression (Fig 3D). AAV2/2 vectors were delivered at 4–4.8 × 10^12^ vg/ml, whereas the AAV2/9 vector was delivered at the highest available titer (2.1 × 10^13^ vg/ml) to achieve the best possible transduction with each vector.

AAV2/2 RNAi + replacement and AAV2/9 RNAi + replacement treatments resulted in similar low-frequency ABR thresholds throughout the study period (Fig 3D). After 12 wk, mid- and high-frequency ABR thresholds of AAV2/2 RNAi + replacement and AAV2/9 RNAi + replacement mice diverged, with improved hearing preservation in mice that received the AAV2/2 vector (Fig 3D). At 8–24 kHz, AAV2/2 RNAi + replacement resulted in 20–40 dB improvement in ABR thresholds relative to AAV2/9 RNAi + replacement, and 40–60 dB improvement in threshold relative to *Bth/+* controls at 20 wk. Conversely, AAV2/2 RNAi + replacement w/ WPRE treatment yielded marginal improvement of ABR thresholds relative to untreated *Bth/+* controls throughout the study period.

At 32 kHz, treatment efficacy was limited regardless of the vector delivered. DPOAE thresholds at 20 wk of age were significantly improved at 32 kHz with AAV2/2 RNAi + replacement relative to *Bth/+* controls (Fig 3E), however, suggesting partial preservation of OHC function at this frequency.

**Figure S4.**
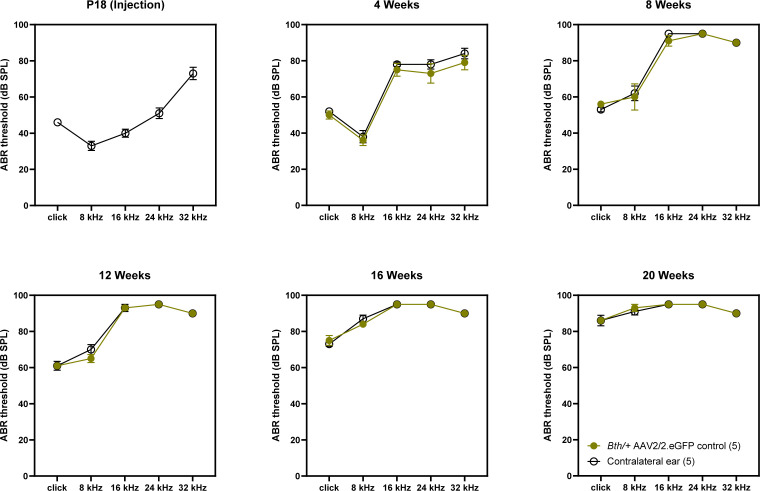
Auditory brainstem response thresholds of *Bth/+* mice with AAV2/2 eGFP control vector. Auditory brainstem response thresholds at 4–20 wk were unchanged in *Bth/+* mice treated with AAV2/2 eGFP vectors. Data are means ± SEM.

Comparing the performance of AAV2/2 RNAi + replacement and AAV2/9 RNAi + replacement with WT and *Bth/+* controls, ABR wave 1 recordings were of shorter latency and greater amplitude in mice that received AAV2/2 relative to *Bth/+* mice that received AAV2/9 or no treatment (Fig 3F–H). Treatment of *Bth/+* mice with AAV2/2.miSafe.eGFP did not result in any changes in ABR thresholds (Fig S4).

### Allele-non-specific silencing and engineered replacement prevent hair cell degeneration

**Figure 4.**
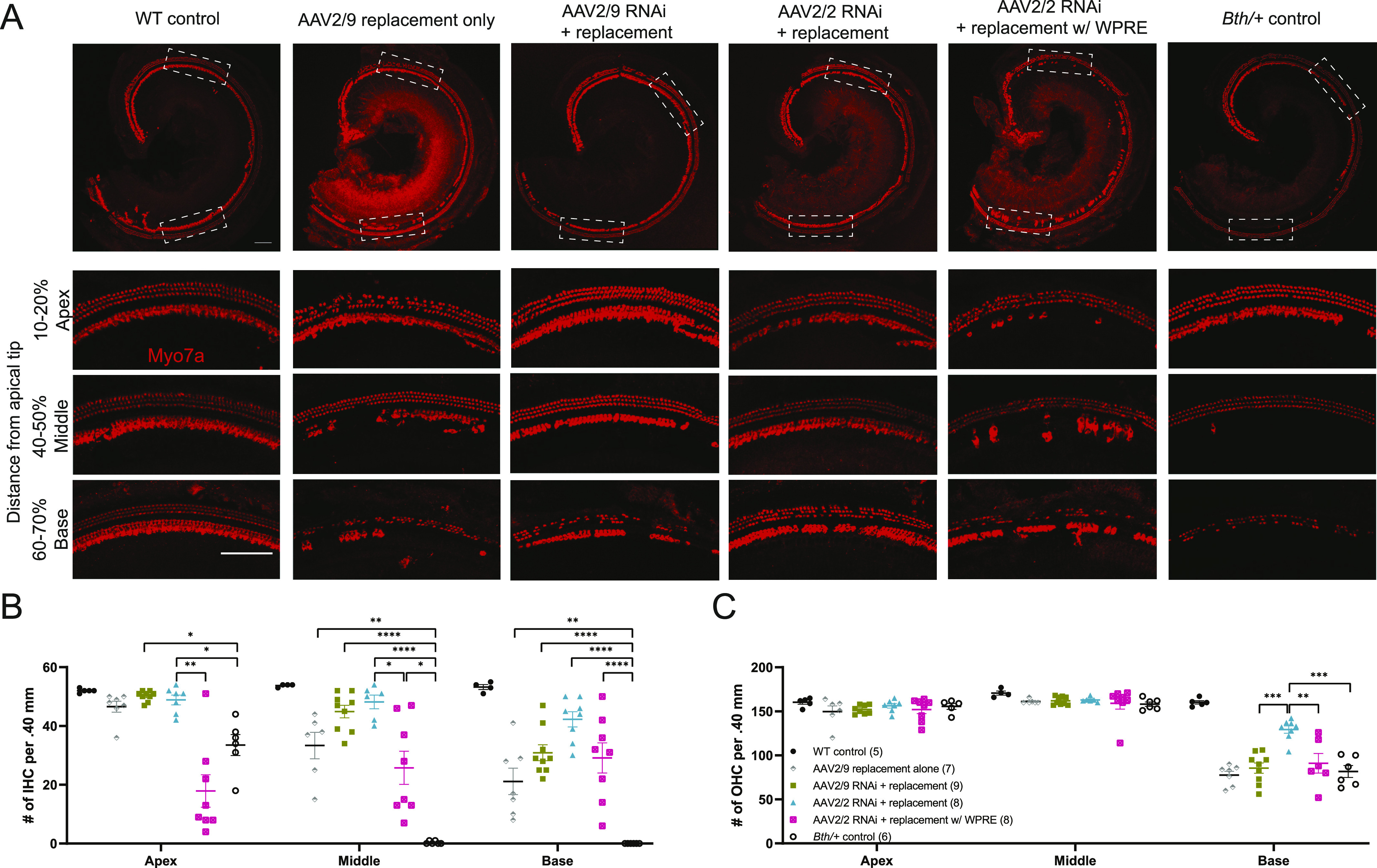
Allele-non-specific RNA interference with engineered replacement enhances hair cell survival up to 20 wk of age. **(A)** Cochlear whole-mount images from WT controls, *Bth/+* controls, AAV2/9 replacement alone, AAV2/9 RNAi + replacement, AAV2/2 RNAi + replacement, and AAV2/2 RNAi + replacement w/WPRE. Cochleae were stained with Myo7a (red) for labeling hair cells. Scale bars represent 100 μm. White dashed boxes represent area of high-magnification image capture for cell counting and comparison of animals treated with viral vectors to *Bth/+* controls. **(B, C)** Quantitative comparison of inner hair cell and outer hair cell survival among all treatment groups. Data are means ± SEM. *N* mice per experimental condition are indicated in parentheses. Statistical analyses are summarized in Tables S5 and S6. ****P < 0.0001; ***P < 0.001; **P < 0.01; and *P < 0.05.

Cochlear hair cells labeled with anti-Myo7a antibody were counted at 20 wk of age, and all tested therapeutic vectors were compared with untreated *Bth/+* controls. Cells were counted in 400-μm segments 10–20% from the apical tip (apex), 40–50% from the apical tip (middle), and 60–70% from the apical tip (base). There was no evidence of hair cell loss in WT controls (Fig 4A, leftmost panel), whereas *Bth/+* controls exhibited ∼40% IHC loss in the apical turn and nearly complete IHC loss in the middle and basal turn. OHC loss was limited in the apical and middle turns, with ∼50% of OHCs lost in the basal turn consistent with previous reports (Fig 4A, rightmost panel) (16).

Table S5 Summary statistical analysis (mean and *P*-values) of inner hair cell counts of animals treated with all tested viral vector therapies versus *Bth/+* controls as summarized in Fig 4B.

Table S6 Summary statistical analysis (mean and *P*-values) of outer hair cell counts of animals treated with all tested viral vector therapies versus *Bth/+* controls as summarized in Fig 4C.

Hair cell survival was improved with the gene therapy construct using both AAV serotypes. With both AAV2/9 RNAi + replacement and AAV2/2 RNAi + replacement, IHCs and OHCs were almost completely preserved throughout the apex and middle turn (Fig 4B). However, only AAV2/2 RNAi + replacement resulted in enhanced OHC survival in the basal turn relative to *Bth/+* controls (Fig 4C). AAV2/2 RNAi + replacement with WPRE generally resulted in poorer HC preservation than other RNAi + replacement vectors (Fig 4B and C).

### Single-cell RNA-Seq implicates increased transgene expression in hair cell degeneration seen with WPRE-containing vectors

**Figure 5.**
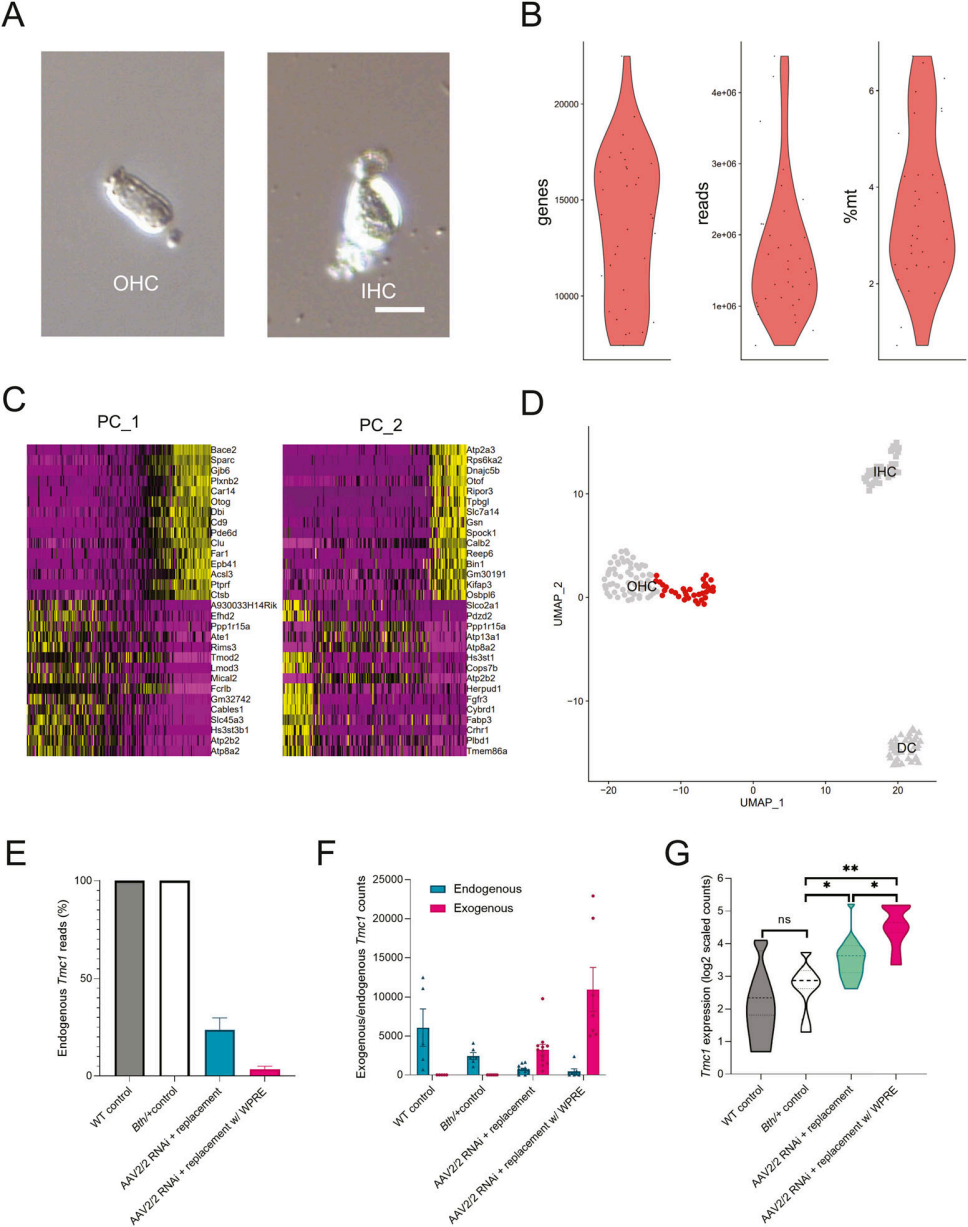
Single-cell RNA sequencing reveals elevated level of transgene expression in treated *Tmc1*^*Bth/+*^ mice. **(A)** Outer hair cells (OHCs) were isolated from WT control, *Bth/+* control, *Bth/+* AAV2/2 RNAi + replacement, and *Bth/+* RNAi + replacement with WPRE mice at P∼60 using manual micropipetting-based isolation. Representative images show dissociated single OHC and inner hair cell. Scale bar represents 20 μm. **(B)** Quality control metrics (*n* unique genes, *n* reads, and percentage of mitochondrial reads) for cells sequenced in this study. **(C, D)** Principal component analysis and unbiased clustering with previously reported dataset of WT cells, untreated OHCs, IHCs, and Deiters’ cells, demonstrating that OHCs of *Tmc1*^*Bth/+*^ animals (red) retained cell-type identity. Expression of highest scoring genes for first two principal components shown as PC_1 and PC_2. **(E)** Estimated percentage of endogenous *Tmc1* transcripts in treated and untreated animals. **(F)** Estimated expression of endogenous and exogenous *Tmc1* in treated and untreated animals. **(G)** *Tmc1* expression was increased in OHCs of *Bth/+* animals that received AAV2/2 RNAi + replacement and AAV2/2 RNAi + replacement with WPRE relative to WT control and *Bth/+* animals (Kruskal–Wallis test, *P* = 0.001, and Dunn’s test: *P* = 0.001 for AAV2/2 RNAi + replacement with WPRE versus *Bth/+* control and AAV2/2 RNAi + replacement with WPRE versus WT control, *P* = 0.04 for AAV2/2 RNAi + replacement versus AAV2/2 RNAi + replacement with WPRE and AAV2/2 RNAi + replacement versus *Bth/+* control, and *P* = 0.045 for AAV2/2 RNAi + control versus WT). *P < 0.05 and **P < 0.01.

Single OHCs were isolated from WT controls, *Bth/+* controls, and *Bth/+* mice that received AAV2/2 RNAi + replacement with or without WPRE, using a manual micropipetting-based isolation strategy based on visual morphologic identification for single-cell RNA sequencing (scRNA-Seq) as previously described (Fig 5A) (25). 34 cells, which passed quality filters (Fig 5B), were used for unbiased clustering (*n* = 7–12 for each group). Cells from our laboratory’s scRNA-Seq dataset (previously reported in Ranum et al) were used to confirm gene expression patterns concordant with the morphologically determined cell-type identity (Fig 5C and D) (25). Read counts of *Tmc1* were adjusted to account for variability in per-cell sequencing depth, and reads containing the entire exogenous or endogenous sequence at the miRNA target site were quantified.

The engineered exogenous *Tmc1* was detected in all OHCs isolated from treated animals, and undetected in untreated OHCs. Moreover, the exogenous allele accounted for 76.4% and 96.5% of *Tmc1* transcripts in AAV2/2.miTmc1D11.mTmc1-injected and AAV2/2.miTmc1D11.mTmc1.WPRE-injected animals, respectively, with estimated knockdown efficiency of 85% among treated animals, confirming successful knockdown and engineered replacement (Fig 5E and F). *Tmc1* expression was equal among OHCs isolated from untreated WT and *Bth/+* control animals (*P* = 0.47). However, mean *Tmc1* expression was increased by 83.6% in animals that received AAV2/2.miTmc1D11.mTmc1, and by 248% in animals that received AAV2/2.miTmc1D11.mTmc1.WPRE (Fig 5G).

## Discussion

In this study, we propose mutation-agnostic RNAi with exogenous engineered replacement for the treatment of genetic hearing loss. Using this strategy, we successfully prevented progression of hearing loss in a murine model of DFNA36. Treated *Bth/+* mice showed dramatic hearing preservation throughout the observation period, with nearly normal thresholds in the best performers. Both click ABR and DPOAE in mice that received AAV2/2 RNAi + replacement were significantly improved relative to untreated mice. Immunohistochemistry at 20 wk showed near-complete IHC preservation and partial OHC preservation, consistent with audiometric findings.

Several authors have reported rescue of autosomal dominant hearing loss models by targeting specific single-nucleotide variants (6, 26), but given the extreme heterogeneity of hearing loss, the widespread translation of variant-specific approaches that do not represent founder mutations is likely to be challenging (27). As a result, there is growing interest in exon- and gene-specific strategies. These approaches have been successfully applied to animal models of retinitis pigmentosa (20, 28), and herein, we demonstrate that mutation-agnostic RNAi combined with gene replacement is effective in treating hearing loss. This approach obviates the need for costly validation of mutation- or exon-specific gene therapies.

Both hearing and hair cell preservation are markedly improved, even relative to earlier studies in our laboratory that leveraged RNAi alone, suggesting that adding gene replacement is more effective than mutation-specific knockdown alone (6). As previous work has established that gene replacement is an appropriate strategy for the treatment of the mouse model of DFNB7/11 (12), the incorporation of gene replacement makes this strategy applicable to both dominant and recessive modes of inheritance. Further study, however, is needed to validate the efficacy of this therapeutic strategy in other murine variant models of DFNA36 and DFNB7/11.

Although a treatment response was observed in click ABR and at 8–24 kHz, there was limited therapeutic efficacy at 32 kHz, a limitation that may reflect the point of injection (P18), at which time *Tmc1*^*Bth/+*^ mice already exhibit severe hearing loss at this frequency (Fig 3C). In earlier studies, our laboratory and others have shown limited efficacy in treatment of the *Tmc1*^*Bth/+*^ model’s severe high-frequency hearing loss, suggesting that damage to HCs may be irreversible (6, 12, 13). Reduced IHC ribbon synapses have also been observed in the basal turn of *Tmc1*^*Bth/+*^ mice as early as P28, suggesting defects in mechanotransduction currents early in life may impair maturation of basal IHC synapses (29). However, transduction efficiency may also be contributory, for although this delivery method achieves robust transduction throughout the cochlea, it is least efficient in OHCs within the basal turn (30, 31).

The cis-regulatory enhancer element WPRE has been used in previous studies delivering both control and gene therapy constructs to the inner ear (12, 13, 31, 32). Surprisingly, in this study, we observed that WPRE-enhanced transduction of eGFP caused severe hearing and hair cell loss in long-term follow-up (Figs 2C and S3). Although eGFP itself has immunogenic and cytotoxic effects (33), we attribute the observed ototoxicity to WPRE-associated increase in eGFP expression, as WPRE-lacking eGFP expression vectors did not cause hearing or hair cell loss (Figs 2B and S3). Similarly, WPRE-containing RNAi + gene replacement vectors were associated with hair cell loss and minimal improvement in ABR thresholds, in spite of the fact that scRNA-Seq data showed an increase of 248% in *Tmc1* expression in OHCs of animals treated with the WPRE-containing vector relative to untreated WT controls (Fig 5G). These findings are consistent with previous reports of cytotoxicity associated with WPRE-induced transgene overexpression (34, 35) and suggest that the level of transgene expressed in target cells is important in optimizing hearing outcomes with gene therapy for *Tmc1*. In contrast, Wu et al recently reported successful rescue in the murine model of *Tmc1*-related autosomal recessive hearing loss, using WPRE-driven *Tmc1* gene replacement delivered by an AAV2/9.PHP.B vector (13). Given the many differences between the present study and the experiments described by Wu et al, the mechanisms underlying these discrepant results are uncertain. Although augmented transgene expression with WPRE is attractive and efficacious in some contexts, our results raise long-term safety concerns for its use in cochlear gene therapy.

There are several limitations to this study. Although this mutation-agnostic approach should be equally applicable to other models of *TMC1*-related hearing loss, further work is necessary to confirm its broad-based applicability. In addition, we have not yet performed studies to determine the mechanism of hair cell death and hearing loss with WPRE-containing vectors, nor to determine the precise level of *Tmc1* expression that is cytotoxic. Whether overexpression of the exogenous *Tmc1* results in cytotoxicity via direct disruption of cation homeostasis and membrane potentials or an alternative mechanism is at play remains to be determined. The number of cells in the scRNA-Seq experiments reported in this study is small and insufficient to thoroughly characterize the transcriptional response to gene therapy or decisively determine in vivo knockdown efficiency of endogenous *Tmc1* by miTmc1D11. Thoroughly cataloguing transcriptional responses to the vectors used in the study herein might reveal the mechanisms underlying failure to rescue high-frequency hearing and the etiology of hearing loss with WPRE-carrying vectors. Lastly, the packaging capacity of AAV is limited to ∼5 kb, which has spurred application of myriad strategies including dual-vector therapies and alternative vectors to deliver large packages to the murine cochlea (36, 37). Simultaneous delivery of an RNAi construct, gene replacement, and associated promoter and regulatory elements is likely to be infeasible for larger hearing loss genes, mandating the use of such techniques.

## Conclusion

In this study, we have proposed a mutation-agnostic approach to cochlear gene therapy leveraging allele-non-specific RNAi and exogenous replacement and demonstrated this strategy’s feasibility for *TMC1*-related hearing loss in a mature murine model of DFNA36. This approach demonstrates superior long-term hearing and hair cell preservation as compared to previously reported RNAi allele-specific strategies or gene replacement alone. Mutation agnosticism allows this strategy to be leveraged for a large group of patients, obviating the need to develop and validate constructs on a variant- or exon-specific basis. In future studies, we plan to apply this mutation-agnostic RNAi strategy to other models of genetic deafness.

## Materials and Methods

### Ethics approval

All experiments were approved by the University of Iowa Institutional Biosafety Committee (IBC; rDNA Committee; rDNA Approval Notice #100024) and the University of Iowa Institutional Animal Care and Use Committee (IACUC; Protocol #06061787) and were performed in accordance with the NIH Guide for the Care and Use of Laboratory Animals.

### Mice

Mice were housed in a controlled temperature environment on a 12-h light/dark cycle. Food and water were provided ad libitum. Isogenic heterozygous Beethoven mice (*Tmc1*^*Bth/+*^) maintained on a C3HeB/FeJ (C3H) background were obtained as a gift from Dr. Karen Steel. Inbred WT C3H mice were obtained from the Jackson Laboratory. Crossbred homozygous *Tmc1*^*Bth/Bth*^ mice were caged with WT C3H mice to generate *Tmc1*^*Bth/+*^ animals. Genotyping was done on DNA from tail-clip biopsies extracted using the Proteinase K method and amplified with forward (5′-CTAATCATACCAAGGAAACATATGGAC-3′) and reverse (5′-TAGACTCACCTTGTTGTTAATCTCATC-3′) primers in a 20-ml volume containing 40 ng of DNA, 24 pmol of each primer, and BioLase DNA polymerase (Bioline) to generate a 376-bp amplification product in *Tmc1*^*Bth/+*^ mice. Amplification conditions included an initial 5-min denaturation at 95°C followed by 35 step cycles of 1 min at 95°C, 1.5 min at 58°C, and 1.5 min at 72°C, with a final elongation of 5 min at 72°C. PCR products were purified and sequenced on an automated sequencer (ABI PRISM 3130xl Genetic Analyzer; Applied Biosystems). An approximately equal number of mice of each sex were used for all experiments, with eight groups receiving seven viral vectors as described in Table 1.

### siRNA design

siRNA sequences were designed using siSPOTR to query possible siRNA-binding positions in mouse *Tmc1* exons 7, 9, 11, 15, and 17 (22). The top 16 siRNA sequences were selected based on their predicted binding affinity and potential off-target score.

### miRNA design

Oligonucleotides were obtained for each siRNA design and cloned into the multiple cloning site (mcs) of the pFBAAVmU6mcsCMVeGFP plasmid obtained from the University of Iowa Viral Vector Core Facility.

### Engineered WT *Tmc1* expression construct

The engineered WT *Tmc1* construct was created by inserting synonymous variants in *Tmc1* at the position of the siRNA-binding site in exon 17 (Fig 1D). Oligonucleotides for this engineered *Tmc1* were cloned into the pFBAAVmU6mcsCMVeGFP plasmid in the position of the eGFP construct, replacing eGFP with engineered *Tmc1* driven by the CMV promoter.

### qRT-PCR

11 of the 16 designed miRNA hairpins were successfully cloned into the multiple cloning site of pFBAAVmU6mcsCMVeGFP expression plasmids obtained from the University of Iowa Viral Vector Core Facility. Ten of the 11 plasmids were successfully evaluated for knockdown performance in an in vitro co-transfection assay using COS-7 cells, a *Tmc1*-deficient cell line, grown in DMEM (Invitrogen, Thermo Fisher Scientific) with 10% FBS at 37°C with 5% CO_2_. In vitro miRNA screening consisted of co-transfection of the above miRNA-expressing plasmids with a mouse *Tmc1*-expressing plasmid pAcGFPm*Tmc1*ex1WT provided by Dr. Andrew Griffith. The transfection mix was made using Lipofectamine 2000 (Invitrogen, Thermo Fisher Scientific) according to the manufacturer’s protocol. RNA was extracted from cells using TRIzol (Invitrogen, Thermo Fisher Scientific).

Initial qRT-PCRs were performed using single biological replicates (technical triplicate) to enable evaluation of higher numbers of miRNAs simultaneously. Top candidates from this initial screening were carried forward and evaluated alongside other top-performing and untested candidate miRNAs in both biological and technical triplicates. Expression levels were assessed in triplicates by qRT-PCR (StepOne Plus, ABI) using intron-spanning *Tmc1* forward (5′-GTTCGCCCAGCAAGATCCTGA-3′) and reverse (5′-GGATGGTAATCTTCCAGTTCAGCA-3′) primer sets and One-step SYBR PrimeScript RT-PCR Kit 2 (Takara–Clontech), normalizing results to β-actin forward (5′-TGAGCGCAAGTACTCTGTGTGGAT-3′) and reverse (5′-ACTCATCGTACTCCTGCTTGCTGA-3′) primer sets. Knockdown was assessed in comparison with a control transfected with pAcGFPmTmc1ex1WT and miRNA-deficient empty vector pFBAAVmU6mcsCMVeGFP. Results were normalized to β-actin with the ΔΔCt algorithm.

### AAV vector production

AAV viral vectors were produced in both AAV2/2 and AAV2/9 serotypes. Four separate plasmid constructs were created and packaged into these serotypes. AAV viral vectors were prepared by the Viral Vector Core at the University of Iowa using a standard triple transfection method in 293FT cells followed by purification in a cesium chloride gradient as previously described (32). Viral titers were AAV2/9.miSafe.mTmc1 at 1.8 × 10^13^ vg/ml, AAV2/9.miTmc1D11.mTmc1 at 2.1 × 10^13^ vg/ml, AAV2/9.miSafe.eGFP at 3.9 × 10^13^ vg/ml, AAV2/2.miSafe.eGFP at 4.2 × 10^12^ vg/ml, AAV2/2.miTmc1D11.mTmc1 at 4.0 × 10^12^ vg/ml, AAV2/2.miTmc1D11.mTmc1.WPRE at 4.8 × 10^12^ vg/ml, and AAV2/2.miSafe.eGFP.WPRE at 5.8 × 10^12^ vg/ml (Table 1). miSafe is an arbitrary sequence with low off-targeting potential, as previously described (14). Virus aliquots were stored at −80°C and thawed before use.

### Animal surgery

A round window membrane combined with canal fenestration (RWM + CF) injection was performed at P16–18, as described previously (30). Mice were anesthetized with an intraperitoneal injection of ketamine (100 mg/kg) and xylazine (10 mg/kg). Body temperature was maintained with a heating pad during the surgical procedure. The left post-auricular region was shaved and cleaned, the site was prepped and draped, and the surgical site was observed under an operating microscope. A post-auricular incision was made to access the temporal bone. After exposing the facial nerve and the sternocleidomastoid muscle (SCM), a portion of the muscle was divided to expose the cochlear bulla ventral to the facial nerve, and the posterior semicircular canal (PSCC) dorsal to the cochlear bulla. A 0.5- to 1.0-mm-diameter otologic drill (Anspach Emax 2 Plus System; DePuy) was used to make a small bullostomy, which was then widened with forceps to visualize the stapedial artery and RWM. A canalostomy was also created in the PSCC with a 0.5-mm-diameter diamond drill; slow egress of perilymph confirmed a patent canalostomy.

1.0 μl of AAV vector with 2.5% fast green dye (Sigma-Aldrich) was loaded into a sharpened borosilicate glass pipette (1.5 mm outer diameter [OD] ∼ 0.86 mm inner diameter [ID]; Harvard Apparatus) pulled with a Sutter P-97 micropipette puller to a final OD of ∼20 μm and affixed to an automated injection system pressured by compressed gas (Harvard Apparatus). Pipettes were manually controlled with a micropipette manipulator. Gene therapy vectors were delivered at titers specified in Table 1. The center of the RWM was gently punctured, and AAV was microinjected into the scala tympani for 3–5 min. Successful injections were confirmed by visualizing the efflux of green fluid from the PSCC canalostomy. After removal of the pipette, the RWM niche was sealed quickly with a small plug of muscle to avoid leakage. The bony defect of the bulla and canal was sealed with small plugs of muscles and Vetbond tissue adhesive (3M). 6-0 absorbable sutures and 6-0 nylon monofilament sutures were used to close the SCM and skin, respectively. Total surgical time ranged from 20 to 30 min. After all procedures, mice were placed on a heating pad for recovery and rubbed with bedding. Pain was controlled with buprenorphine (0.05 mg/kg) and/or flunixin meglumine (2.5 mg/kg) for at least 2 d. Recovery was closely monitored daily for at least 5 d post-operatively. All animals were operated on by one surgeon (Y.I.).

### Auditory testing

ABRs were recorded as described previously (30). All mice were anesthetized with an intraperitoneal injection of ketamine (100 mg/kg) and xylazine (10 mg/kg). All recordings were conducted from both ears of all animals on a heating pad using electrodes placed subcutaneously in the vertex and underneath the left or right ear. Clicks were square pulses 100 ms in duration, and tone bursts were 3 ms in length at distinct 8-, 16-, 24-, and 32-kHz frequencies. ABRs were measured with BioSigRZ (Tucker–Davis Technologies) for both clicks and tone bursts, adjusting the stimulus levels from 90 dB SPL to 5 dB SPL in a 5 dB decrement. Electrical signals were averaged over 512 repetitions. ABR thresholds were defined as the lowest sound level at which a reproducible wave 1 could be observed. For transduction efficiency analysis, ABRs were measured at 4 and 10 wk; for gene therapy experiments, they were measured at 4-wk intervals for 20 wk. Responses from either the left ear in WT or untreated *Tmc1*^*Bth/+*^ mice, or the contralateral ear that did not undergo surgery were used as controls.

Distortion product otoacoustic emissions (DPOAEs) were measured after ABR recording. DPOAEs were measured at the following center frequencies (Fc): 4, 8, 16, and 32 kHz. Primary signals (F1 and F2) were calculated using Fc where F1 is Fc ×0.909 and F2 is Fc ×1.09. F1 and F2 were generated from two separate MF1 speakers at an intensity level from 80 to 20 dB SPL in a 10 dB decrement. The level of two primary tones was set equal (L1 = L2). DPOAE threshold was defined as the lowest level of F2 where the DPOAE amplitude at 2F1 – F2 was at least 3 dB above the average level of noise floor.

### Immunohistochemistry, cell counts, and transduction efficiency analysis

All injected and non-injected cochleae were harvested after animals were euthanized by CO_2_ inhalation. Temporal bones were locally perfused and fixed in 4% PFA for 2 h at 4°C, rinsed in PBS, and stored at 4°C in preparation for immunohistochemistry. Specimens were visualized with a dissection microscope and dissected for whole-mount analysis. In all cochlear whole mounts, GFP was detected by intrinsic fluorescence. After infiltration using 0.3% Triton X-100 for 30 min and blocking with 5% normal goat serum for 30 min, tissues were incubated with rabbit polyclonal Myosin-VIIA antibody (#25-6790; Proteus Biosciences) diluted 1:200 in PBS for 1 h. Subsequently, fluorescence-labeled goat anti-rabbit IgG Alexa Fluor 568 (#A-11036; Thermo Fisher Scientific) at 1:500 dilution was used as a secondary antibody for 30 min. Specimens were mounted using ProLong Diamond Antifade Mountant with DAPI (#P36965; Thermo Fisher Scientific) and observed with a Leica TCS SP8 or Leica DMi8 confocal microscope (Leica Microsystems). Cell counts and transduction efficiency analysis were performed as described previously (30, 31). Z-stack images of whole mounts were collected at 10×–20× on a Leica SP8 confocal microscope. Each turn of the cochlea was analyzed as follows: 10–20% from apical tip (apex); 40–50% from apical tip (middle); and 60–70% from apical tip (base), corresponding to approximate frequencies of 8, 16, and 24–32 kHz, respectively (38). Maximum-intensity projections of Z-stacks were generated for each field of view, and images were prepared using LAS X (Leica Microsystems) to meet equal conditions. IHCs and OHCs were counted per 400-μm cochlear sections for each turn in each specimen with ImageJ Cell Counter (NIH Image). For transduction efficiency, IHCs and OHCs with positive eGFP and with immunostaining for Myo7a were counted; the total numbers of HCs and eGFP-positive HCs were summed and converted to a percentage.

### Single-cell RNA sequencing

scRNA-Seq was performed as previously described, with minor modifications (25). After euthanasia via CO_2_ at P57–P69, murine temporal bones were harvested and transferred to a culture dish containing ice-cold 1× DPBS for visualization under a dissecting microscope (Model M165FC; Leica Microsystems). Bone covering the apical turn was removed with #5 forceps (Fine Science Tools) to access the membranous labyrinth, which was transferred to a 1.5-ml tube containing Accumax (Innovative Cell Technologies) for digestion at RT for 5 min. Gentle pipetting was used to mechanically dissociate single hair cells, and the disrupted tissue and digestion buffer were then transferred to a glass microscope slide (Superfrost Plus 25 × 75 × 1.0 mm; Thermo Fisher Scientific). Hair cells were visualized on an inverted microscope (Model DMI3000B; Leica Microsystems) equipped with 20× and 40× DIC objectives and a 3D micromanipulator (Model MN-153; Narishige) driving a pulled glass micropipette attached to a nitrogen gas–powered pico-injector (Model PLI-100; Harvard Apparatus) used to control aspiration pressure. A wash slide of 1× DPBS was immediately adjacent. Candidate cells were identified by scanning the field of view and aspirated into the first glass micropipette in a slow and controlled manner. Cells were transferred to the adjacent 1× DPBS wash slide and washed to remove extracellular debris, then reaspirated using a second clean glass micropipette. The washed cell was expelled into a 0.2-ml tube containing RNase inhibitor–containing lysis buffer (Takara Bio). The tubes were briefly centrifuged and flash-frozen on dry ice, then stored at −80°C before RT–PCR and amplification using Smart-Seq HT (Takara Bio) according to the manufacturer’s protocol. Illumina library preparation was performed with Nextera XT (Illumina) before equimolar pooling for sequencing on a single lane of an Illumina NovaSeq 6000 using 250-bp paired-end read chemistry.

### Illumina sequencing data analysis

Raw sequence FASTQs were pseudo-aligned and transcripts quantified by Kallisto 0.48.0 (39) using the Ensembl 106 *Mus musculus* GRCm39 transcriptome and 100 bootstrap samples. Transcript counts were summarized as per-cell gene counts using tximport 1.24.0 (40). Expression patterns were confirmed to be concordant with OHCs by unbiased clustering analysis using Seurat 4.1.1 (41). First, gene counts were combined with counts computed in the same way for known Deiters’ cells, IHCs, and OHCs at P15 from Ranum et al (25). Genes were filtered to those present in at least three cells, and cells were filtered for quality to those with at least 3,000 such genes expressed and mitochondrial reads ≤7%. Normalization, feature selection, and scaling were performed per defaults. Cells were then clustered using the first three principal components and resolution 0.05—parameters empirically chosen to form three cell-type clusters. Finally, cluster membership for each cell in this study was examined and cells belonging to the cluster with all the known outer hair cells were retained for subsequent analyses.

### Statistical analysis

Two groups were compared using Welch’s *t* test. For comparisons between more than two groups, one-way ANOVA was performed followed by post hoc analysis with Tukey’s multiple comparisons test; when SDs were significantly different by Bartlett’s test, Welch’s ANOVA with post hoc Dunnett’s T3 multiple comparisons test was performed. *P* < 0.05 was considered statistically significant. Statistical analysis of *Tmc1* expression was performed using R 4.2.0 (42) and the FSA 0.9.3 R package Dunn’s test function (43). To estimate endogenous and exogenous *Tmc1* expression and knockdown efficiency in vivo, reads containing exact matches for the endogenous (GACGATCATGTACAGGACCGG) or the exogenous (CACTATCATATATAGTACTGG) sequence, or the reverse complement thereof, at the RNAi-binding site were counted to estimate the percentage of endogenous and exogenous reads in all cells containing reads at the siRNA-binding site. Per-cell *Tmc1* expression was then multiplied by these percentages to generate a rough estimate of the expression of exogenous and endogenous *Tmc1* on a per-cell basis. *Tmc1* expression levels were compared using the Kruskal–Wallis test followed by post hoc analysis using Dunn’s test with the Benjamini–Hochberg adjustment.

## Data Availability

RNA-sequencing data reported in this study are available at https://umgear.org/p?s=c65e0b22 and will be submitted to the Gene Expression Omnibus database. Software resources used are summarized in the Reagents and Tools Table. Requests for additional data will be distributed upon request.

## Supplementary Material

Reviewer comments
